# Investigation of Cavity Enhanced XEOL of a Single ZnO Microrod by Using Multifunctional Hard X-ray Nanoprobe

**DOI:** 10.1038/s41598-018-36764-8

**Published:** 2019-01-18

**Authors:** Bi-Hsuan Lin, Xiao-Yun Li, Dai-Jie Lin, Bo-Lun Jian, Hsu-Cheng Hsu, Huang-Yen Chen, Shao-Chin Tseng, Chien-Yu Lee, Bo-Yi Chen, Gung-Chian Yin, Ming-Ying Hsu, Shih-Hung Chang, Mau-Tsu Tang, Wen-Feng Hsieh

**Affiliations:** 1 National Synchrotron Radiation Research Center, Hsinchu, 30076 Taiwan; 2 Department of Photonics, National Cheng Kung University, Tainan, 701 Taiwan; 3 Department of Photonics and Institute of Electro-Optical Engineering, National Chiao Tung University, Hsinchu, 30010 Taiwan

## Abstract

The multifunctional hard X-ray nanoprobe at Taiwan Photon Source (TPS) exhibits the excellent ability to simultaneously characterize the X-ray absorption, X-ray excited optical luminescence (XEOL) as well as the dynamics of XEOL of materials. Combining the scanning electron microscope (SEM) into the TPS 23A end-station, we can easily and quickly measure the optical properties to map out the morphology of a ZnO microrod. A special phenomenon has been observed that the oscillations in the XEOL associated with the confinement of the optical photons in the single ZnO microrod shows dramatical increase while the X-ray excitation energy is set across the Zn K-edge. Besides having the nano-scale spatial resolution, the synchrotron source also gives a good temporal domain measurement to investigate the luminescence dynamic process. The decay lifetimes of different emission wavelengths and can be simultaneously obtained from the streak image. Besides, SEM can provide the cathodoluminescence (CL) to be a complementary method to analyze the emission properties of materials, we anticipate that the X-ray nanoprobe will open new avenues with great characterization ability for developing nano/microsized optoelectronic devices.

## Introduction

In order to integrate a compact photonic system or module with enhanced properties and functions, one of the key ways is to miniaturize the light sources^1,2^. The advantage of having wide direct band-gap (3.37 eV) and large exciton binding energy (60 meV) at room temperature (RT), ZnO has attracted much attention to be the UV emitters in recent years. Many nano/microsized optoelectronic devices have been investigated by using various ZnO nano/microstructures such as microrods^3^, microdisks^4^, nanoribbons^5^, nanowires^6^, and nanoflag^7^. Especially, RT microcavity lasing^8,9^ has been achieved in use of ZnO nano/microstructures and gained significant research interest. Beside to grow high quality nano/microstructures, the ability to characterize the optical, structural and electronical properties of nano/microstructures is important for the applications of nano/microsized optoelectronic devices. The whispering gallery mode (WGM) microcavity made of a hexagonal ZnO microrod is one of the most important type of optical cavities that have been studied widely by using UV lasers as the pump sources^9–12^. However, it is still rare to probe in the hard X-ray energy (multi-keV) range to characterize the optical properties of a single ZnO microrod with high spatial resoliution. Based on the advantage of using synchrotron source with continuous and tunable X-ray energy, we have used unfocused hard X-ray excited optical luminescence (XEOL) with X-ray energy set across the Zn K-edge and observed peculiar near-band-edge (NBE) emission from polar^13^ and non-polar^14^ ZnO wafers. The XEOL spectroscopy has been reported completely by Sham *et al*.^15–17^ as a powerful tool to characterize the optical properties of the materials. Martinez-criado *et al*.^18^ have also elucidated the benefits of using synchrotron based probes to study the nano/microstructures, in which a single probe can be used to characterize simultaneously many properties, for example, the X-ray fluorescence (XRF) and the X-ray absorption spectroscopy (XAS) can be used to measure the chemical trace of the element distribution; the X-ray diffraction (XRD) can be used as a structural probe; and the XEOL and time-resolved XEOL (TR-XEOL) can be applied for non-destructive probing the optical properties and dynamics of luminescence. *Salomon, et al*.^19^ also exhibited the capabilities of nanoprobe beamline to investigate the induced local lattice polarity in single GaN wires. Based on our previous XEOL experience, we have completed a X-ray nanoprobe beamline in Taiwan Photon Source (TPS), thus, in this report, we will not only show the capabilities of our X-ray nanoprobe beamline in TPS, but also apply the nano-focused X-ray beam to study the emission properties of a single ZnO microrod by the XEOL and the TR-XEOL for determining the decay time of XEOL from this single ZnO microrod.

## Methods

**Figure 1:**
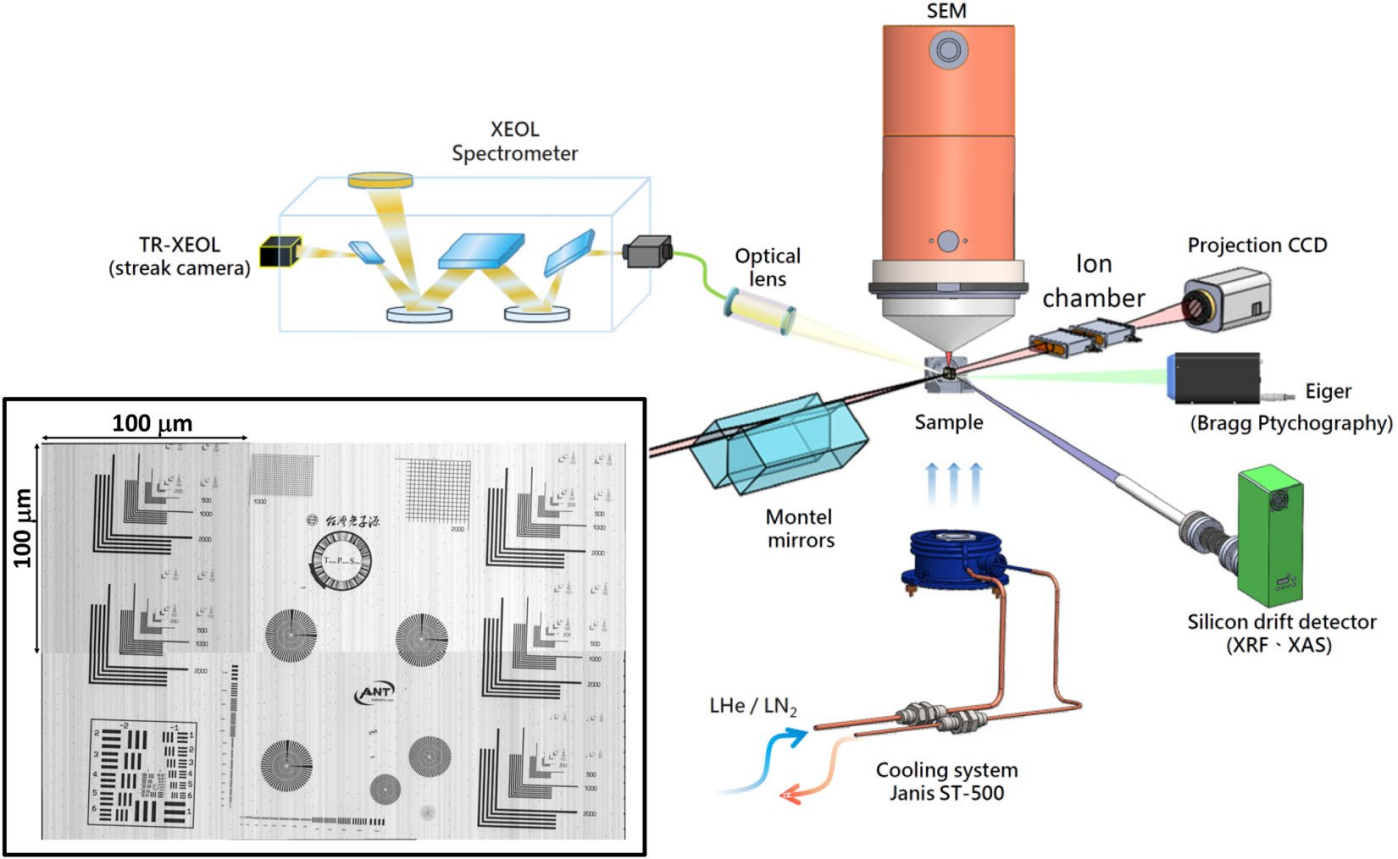
Multifunction hard X-ray nanoprobe at TPS 23A of the Taiwan Photon Source (TPS). The inset is the contrast image by using ion chamber.

**Figure 2:**
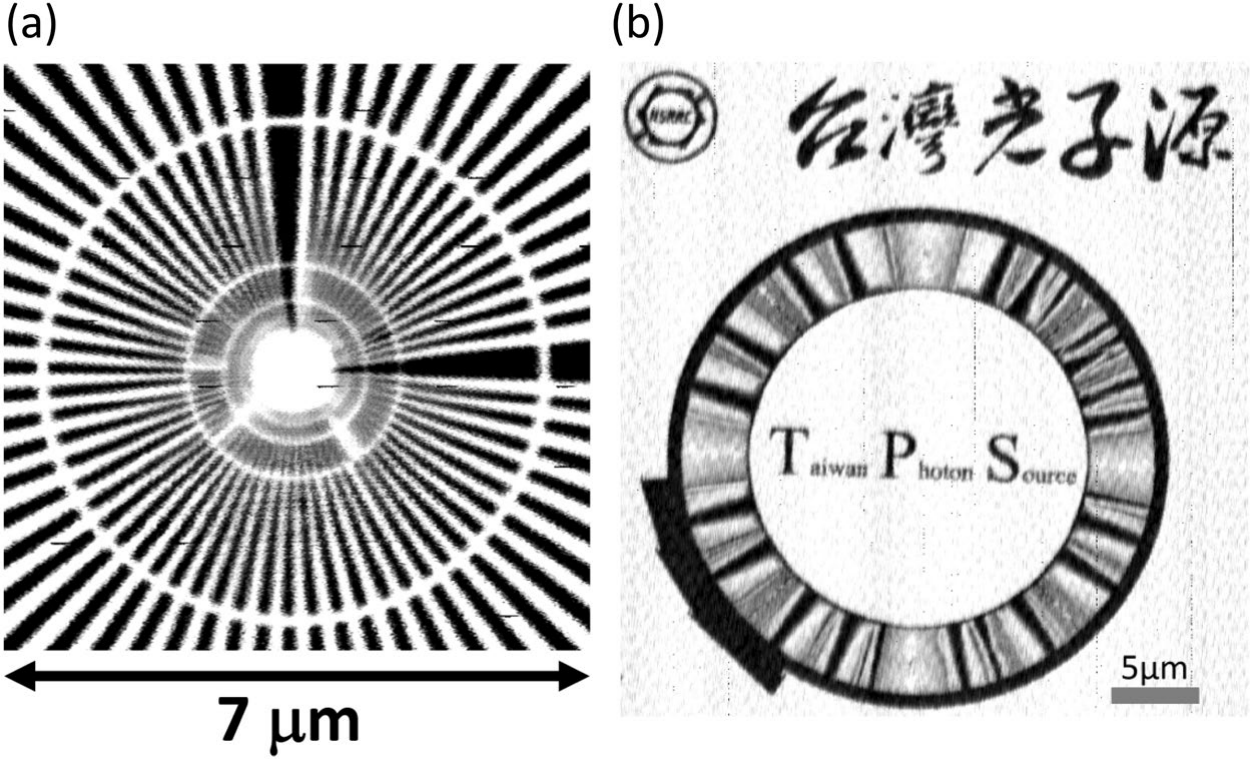
(**a,b**) are the contrast image by using ion chamber to measure the X-ray beam size.

The TPS 23A X-ray nanoprobe is a new constructed beamline at Taiwan Photon Source at National Synchrotron Radiation Research Center (NSRRC) in Taiwan. Figure 1 shows the schematic of the experimental setup of this nanoprobe end-station. This beamline provides a hard X-ray energy ranged from 4 to 15 keV, and the excited X-ray energy was tuned and selected by using a double Si(111) crystal monochromator with a typical resolution (Δ*E*/*E*) of ~2 × 10^−4^. The specification of Motel mirrors^20^ and the detail design of this nanoprobe can be found in the report of Yin *et al*.^21^. The main functions of this beamline include XEOL, TR-XEOL, XRF, XAS, CL, XRD and Bragg ptychography^22^ with high spatial and temporal resolution so that in a single probe one can simultaneously obtain the optical, compositional and structural information. The SEM was equipped in a high vacuum chamber (1 × 10^−6^ torr) with a load-lock system to quickly transfer the samples between the main chamber and the preparation chamber. The information at the precise location and morphology of the nano/microstructure can be obtained easily and quickly through this SEM system. The sample compartment is designed to equip with a cryostat system that maintains at temperature range of 10 K~300 K for the XEOL and TR-XEOL measurements. The ion chamber system provides not only quick X-ray energy calibration using standard element foils but also contrast image mapping to measure the X-ray beam size. The inset of Fig. 1 is the contrast image mapping by using focused ion beam, and the standard sample is made by 550 nm thick Au. The image was composed by six of 100 μm x 100 μm images, because of the traveling ranges of fine and course flexure piezo sample stages are 100 μm and 25 mm, respectively. Figure 2(a,b) are the images in the standard sample, which can be found in the inset of Fig. 1. The X-ray beam size can be examined through the spoke pattern of Fig. 2(a) and the beam size is determined to be about 60 nm since the third circle lines of the spoken pattern can be clearly resolved. The Chinese calligraphy in the contrast image of Fig. 2(b), which means “Taiwan Photon Source”, is from a famous ancient Chinese literator and poet, Tung-Po Su, who lived from AD 1037 to 1101.

The X-ray energy for the XAS and XEOL measurements was set across the Zn K-edge (9.659 keV) with the photon flux estimated to be around 10^9^∼10^10^ photons/s. The Zn K-edge absorption spectra of single ZnO microrod were measured by a silicon drift detector (SDD), Vortex-ME4, HITACHI. The XEOL spectra were collected by an optical fiber and a spectrometer (iHR550, HORIBA) with a liquid nitrogen cooled charge-coupled device (CCD, BIUV Symphony II) having 2048 × 512 pixels. A switching mirror was equipped in the iHR550 spectrometer with a photomultiplier tube (PMT)^23^, which can be used to perform the CL mapping images. The TR-XEOL was measured the synchrotron source with repetition frequency of 499.665 MHz and 30 ps pulse duration, which gives the available time scale between 30 ps~2 ns for determining luminescence lifetime. The TR-XEOL spectra were also collected by a fiber, which is coupled to a spectrometer (iHR320, HORIBA) with Hamamatsu C10910 streak camera and M10913 slow single sweep unit. Due to the maximum sweep repetition frequency of the slow single sweep unit is 4 MHz, the repetition frequency of TR-XEOL was operated in 3.469 MHz (499.665 MHz /144). ZnO microrods were grown on Si substrates by chemical vapor deposition^24^. ZnO and graphite powders were mixed and loaded in an alumina boat serving as source material. Two cleaned Si substrates were placed face down above a mixture of ZnO and graphite powders with a gap of about 5 mm on an alumina boat located at the center of the quartz tube furnace. The furnace was heated to 1050 °C about 50 minutes and maintained at that temperature for 1 hour. ZnO microrods were mostly found on the edge of the Si substrates.

## Results and Discussion

**Figure 3:**
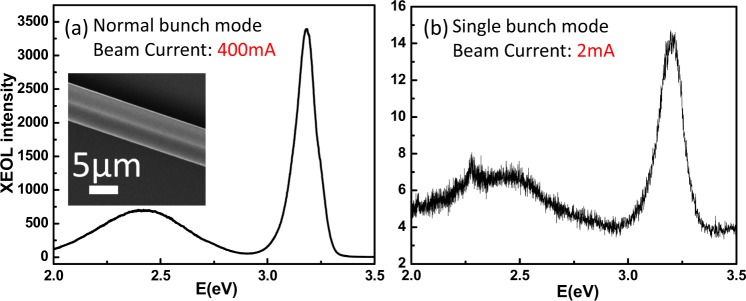
The comparison of the XEOL spectra between normal (**a**) and single (**b**) bunch modes. The measured single ZnO microrod is shown in the inset of (**a**).

In order to have excellent performance on great characterization ability, a serious of experiments were designed to examine the capabilities of this new beamline. The comparison of the XEOL spectra of single ZnO microrod between normal and single bunch mode^25^ are shown in Fig. 3(a,b), respectively. The exposure times of the both modes are the same with only 1 second. The beam current and repetition rate of normal bunch mode are 400 mA and 499.665 MHz, respectively, and those of single bunch mode are 2 mA and 578 KHz. The XEOL spectra in both modes show clearly and typically the emission properties of ZnO with NBE and defect band. Although the beam current of single bunch mode is 200 times of magnitude smaller than that of normal bunch mode, we also can obtain the good XEOL spectrum of single ZnO microrod in the single bunch mode. The reason of measuring in the single bunch mode is that the longer time scale (30 ps~1.72 μs) can be used for the materials which having longer decay time^26^. The disadvantage of single bunch mode is the lower beam current, so we are going to develop the hybrid bunch mode^27^ to satisfy simultaneously the requirements of photon flux and decay time.

**Figure 4:**
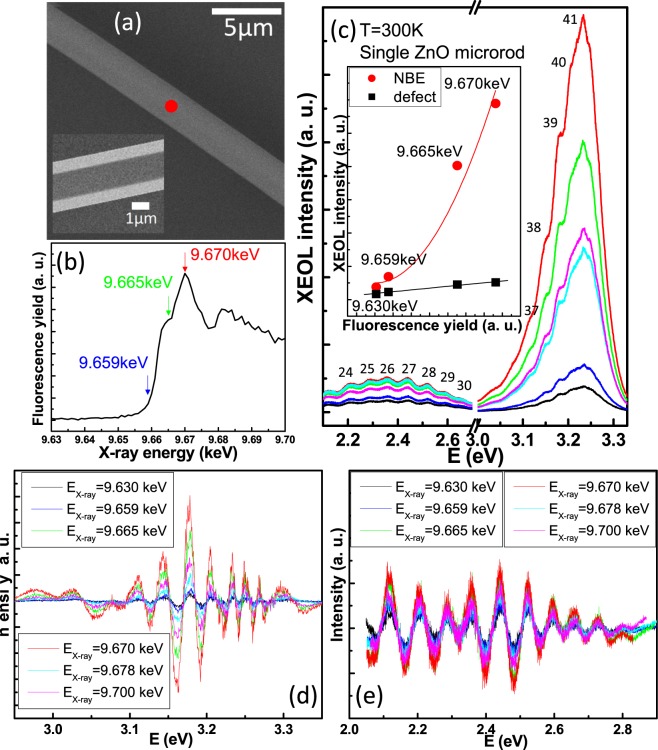
The SEM image (**a**) and the Zn K-edge X-ray absorption (**b**) of a single ZnO microrod with diameter of about 2.8 μm and its XEOL spectra (**c**) under the excitation X-ray energy set across the Zn K-edge measured at room temperature. The X-ray focused spot is marked by the red point. The inset of (**c**) shows the NBE (red circle) and defect band (black square) emission intensities versus the fluorescence yield of XAS. The extracted oscillations of the NBE and defect emissions in (**c**) were shown in (**d,e**), respectively.

In use of the TPS 23A nanoprobe, the X-ray beam can be focused to a spot of ~60 nm × 60 nm on a single ZnO microrod. Figure 4 exhibits the advantage of this single probe beamline including the functions of SEM, XAS and XEOL. The position and morphology of the single ZnO microrod with diameter of about 2.8 μm can be easily measured by SEM which is shown in Fig. 4(a). The inset of Fig. 4(a) was cross-checked with in-house SEM to examine the hexagonal shape of the single ZnO microrod. The XAS of the focus point marked in Fig. 4(a) with the X-ray energy being set across the Zn K-edge (9.659 keV) is shown in Fig. 4(b). The RT XEOL spectra of the single ZnO microrod under the excitation X-ray energy set across the Zn K-edge are shown in Fig. 4(c). We found that not only the emission intensity of the ZnO NBE increases rapidly but also no blue shift in XEOL peak as the excitation X-ray energy is set across the Zn K-edge, and the largest emission intensity is occurred at the X-ray energy of 9.670 keV. The NBE emission intensity of the single ZnO microrod indicates increasing more rapidly than that of the defect band at around 2.4 eV. The branching ratios of I_NBE_/I_defect_ with the six X-ray energies at 9.630, 9.659, 9.665, 9.670, 9.678 and 9.700 keV are estimated about 2.18, 3.01, 8.96, 11.35, 4.93 and 7.37, respectively. The inset of the Fig. 4(c) shows the NBE (red circles) and defect band (black squares) emission intensities versus the fluorescence yield of XAS. As compared with the linearly increasing intensity of defect band emission, the intensity of NBE emission shows obviously non-linear increasing with the fluorescence yield of XAS. Similar arising behaviors have also been observed by Wang *et al*.^28^ in the ZnO nanowire arrays with soft X-ray energy across the O K-edge and Zn L_3,2_-edge. The difference in our report is that we found all the XEOL spectra of the single ZnO microrod show obviously oscillatory feature superimposing over a broad spontaneous emission band that implies resonance effect with optical feedback by certain cavity modes or WGMs^12^ to cause stimulated process to take place.

In order to pursuit more on the origins of the resonance effect^29^ in the NBE and defect emission bands in Fig. 4(c). We fitted each of the spectra of Fig. 4(c) by a Gaussian function, then subtracted the measured emission spectra by the fitting results to obtain the oscillation spectra as shown in Fig. 4(d,e). It can be seen that the oscillation spectra of defect emission appear not only linearly proportional to the X-ray absorption but also almost a periodic function with almost equal energy spacing of 74 meV. However, those of the NBE emission show not only clearly enhancement (nonlinear) in oscillating amplitude with the X-ray absorption similar to that of broad NBE emission but also the closer energy spacing as increasing the XEOL photon energy. According to the formula^8^: ${\rm{\Delta }}{\rm{\lambda }}={\lambda }^{2}/[L(n-\lambda dn/d\lambda )]$ and assuming dispersionless (*dn*/*dλ* = 0 with a constant refractive index of *n* = 2.2 at off-exciton resonance around 2.4 eV, we calculated the cavity length (or microrod diameter) as *L* = 7.3 μm, which corresponds to diameter *D* = 2.8 μm close to the measured one by SEM. Furthermore, the mode number can be also calculated by the WGM equation^8,30^:

1 $$N=\frac{3\sqrt{3}nD}{2\lambda }-\frac{6}{\pi }\ast {ta}{{n}}^{-1}(\frac{1}{n}\sqrt{3{n}^{2}-4})$$

where N is the mode number, n is the refractive index of ZnO^30^, *D* is the diameter of the microrod. The numerically calculated modes match with the experimentally observed peak positions very well, as indicated in Fig. 4(c). Therefore, we can conclude that the WGM resonance cavity can be formed in this single ZnO microrod. By plotting the dispersion curve *ω*(*k*) and assuming it is linear around 2.4 eV, we obtained the data points of oscillation peaks (and valleys) are located on the lower branch of exciton polariton (not shown here)^31^. These demonstrations indicate that the X-ray nanoprobe can excite large enough number of excitons to enable exciton-exciton scattering and/or to provide gain to the WGM resonance in the single ZnO microrod.

Compared with the UV laser pumping, the advantages of nanoprobe XEOL are the X-ray excitation energy can be tuned across the specific K-edge of element to excite the core electrons leaving core holes and photoelectrons and the nanoprobe can provide huge X-ray dose into the specific area of the sample. The spurted emission intensity at the resonance X-ray energy may be caused not only by WGM microcavity but also by the strong peak power density resulting from the nano-focused beam. The peak power density of TPS-23A X-ray nanoprobe is about 3 × 10^6^ W/cm^2^ under the experimental conditions of a synchrotron source having 500 MHz repetition rate with 10^9^ photons/s and 30 ps pulse width being focused to a spot size of about 60 nm × 60 nm. Compared with the peak power density in the unfocused X-ray beam of BL 07A at Taiwan Light Source^14^, the peak power density of nano-focused X-ray beam is about 5 orders of magnitude larger than that of unfocused X-ray beam. It means that the gain of nano-focused X-ray beam is about 5 orders of magnitude larger than that of unfocused X-ray beam with gain length of 60 nm for nanoprobe versus 1 *μ*m for unfocused one.

The sharp corners and so the higher Q-factor of the hexagonal microcavity in ZnO microrod^32^ is another factor to influence the WGM lasing. So, a better microcavity structure of ZnO microrod is able to show more obvious WGM lasing with resonance X-ray energy excitation. The positive-edge jump behavior of Fig. 4(c) has been observed in our previous report^14^ and by Armelao *et al*.^33^ in the ZnO nanoneedle sample. It is attributed to the NBE emission is proportional to the absorption coefficient. According to the elucidation of Martinez-Criado *et al*.^34^ used the hard X-ray Ga K-edge for GaN, the positive-edge jump suggests that the high energy electrons excited from inner core states by X-ray across Zn K-edge region contribute more to the optical luminescence than those from outer core states.

**Figure 5:**
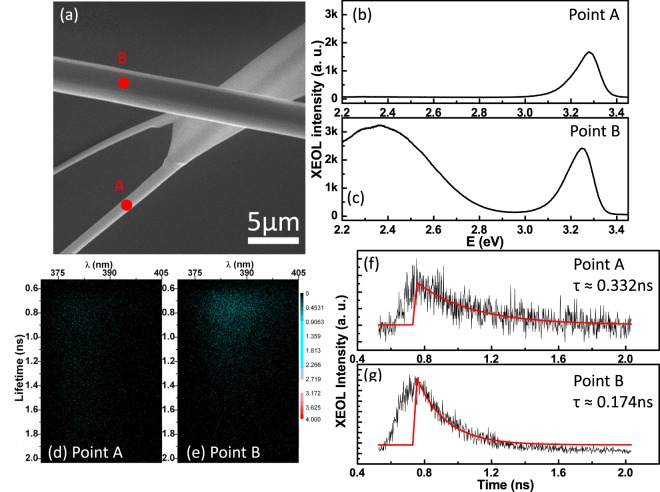
The XEOL spectra as the X-ray being focused at (**b**) Point A and (**c**) Point B, shown as the red markers at the SEM image (**a**) of the ZnO microrods. The corresponding TR-XEOL streak images of (**d**) Point A and (**e**) Point B and the fitting the decay times from the TR-XEOL traces of (**f**) Point A in (**d,g**) Point B in (**e**), respectively.

Beside the emission properties, the dynamics of luminescence was also studied under the TPS 23A nanoprobe. Understanding the luminescence process will improve the internal quantum efficiency or reduce the non-radiative losses for the design of optoelectronic devices. The high spatial resolution of the TPS 23A nanoprobe can be used to investigate the dynamics optical phenomena under the advantage of synchrotron source with the ability of high temporal resolution. Having SEM, one can easily and quickly choose points of interest on the ZnO microrods to study the dynamics of XEOL. Figure 5(a) shows a SEM image of a straight ZnO microrod across a dendritic-like ZnO microrod. The red points A and B are the measured positions of XEOL. Their corresponding XEOL spectra were plotted in Fig. 5(b,c). The point A on the dendritic-like ZnO microrod shows mainly NBE emission with much less defect band emission than that of the point B of the straight ZnO microrod. Similar emission behaviors have also been observed in the ZnO with nanoflower, nanocomb and nanoflag structures^7,35^. The time-resolved XEOL taken with a streak camera at points A and B were shown in Fig. 5(d,e), respectively. The advantage of using streak camera is that simultaneously obtain the decay lifetimes at different emission wavelengths that has been more detail described by Ward *et al*.^36^. Fitting the spectral integrated (370 nm~405 nm) time traces of points A and B by a single exponential function shown in Fig. 5(f,g), we obtained the decay lifetimes for point A ($\tau \approx 0.332\,ns$) is almost 2 times longer than that of point B ($\tau \approx 0.174\,ns$). The results suggest that the shorter luminescence lifetime of point B than that of point A is attributed to carriers being trapped to the defect states to show apparent the defect band emission in Fig. 5(c). We also examined the life time of defects light should be longer than 2 ns due to the limitation of experimental condition. A special experimental condition such as single bunch mode is needed to measure the life time of defect light. This observation suggests that with this advanced instrumentation capable of study an individual nanostructure, we are getting closer to resolve the morphology and crystallinity dependent energy transfer (X-ray to optical) in nanostructures.

**Figure 6:**
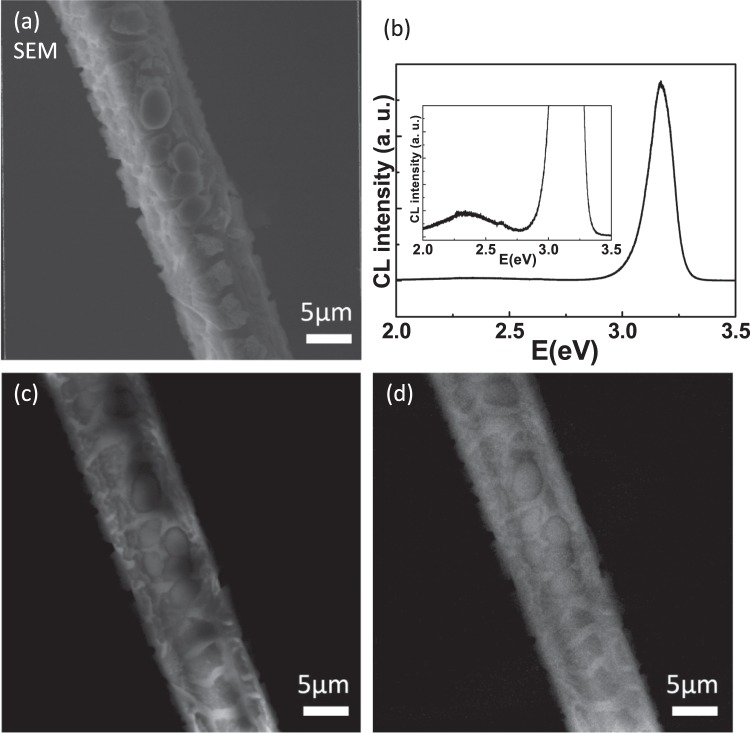
(**a**,**b**) show the SEM image and CL spectra of a scaly ZnO microrod, respectively. The defect band emission can be observed in the inset of (**b**). The CL mapping images of NBE and defect emission are show in (**c,d**), respectively.

Combining the SEM into the TPS 23A end-station can not only find the sample position easily and quickly, but also can apply the electron source to conduct the CL analysis. Figure 6(a,b) show the SEM image and CL spectra of a scaly-like ZnO microrod, respectively. The defect band emission can be observed clearly in the inset of Fig. 6(b). The NBE and defect band emissions of the scaly-like ZnO microrod are located about 3.2 eV and 2.3 eV, respectively. Then, the CL mapping images can be used to analyze the emission distribution of NBE and defect band, which were shown in Fig. 6(c,d), respectively. The properties of emission distribution of the scaly-like ZnO microrod can be analyzed quickly through comparing the CL mapping images of NBE and defect band.

## Conclusions

Based on the advantage of TPS 23A X-ray nanoprobe equipped with a SEM system, we have the ability to use single probe to simultaneously characterize the morphology, the X-ray absorption, the near-band edge emission, and the dynamic of luminescence in a single ZnO microrod, respectively, by XAS, XEOL and TR-XEOL with high position resolution. The SEM system applied not only a good solution to easily and quickly obtain the position of the ZnO microrods, but also the electron source to perform the CL mapping technique. SEM is complementary with X-ray analysis, and the materials properties can be analyzed more completely by using their advantages. The X-ray nanobeam provides large peak power density for us to observe the cavity enhanced phenomenon that the amplified spontaneous emission of the single ZnO microrod can be exhibited at the resonance excitation energy. Besides having the excellent spatial resolution, the synchrotron source also gives a short enough temporal pulse to investigate the dynamics of luminescence processes with the information of decay lifetime at different emission wavelengths can be simultaneously recorded in a streak image. We anticipate that the TPS 23A X-ray nanoprobe will contribute a great characterization ability for developing the nano/microsized optoelectronic devices.
